# Analysis of autosomal dominant genes impacted by copy number loss in 24,844 fetuses without structural abnormalities

**DOI:** 10.1186/s12864-022-08340-y

**Published:** 2022-02-02

**Authors:** Lin Chen, Li Wang, Daishu Yin, Feng Tang, Yang Zeng, Hongmei Zhu, Jing Wang

**Affiliations:** 1 Department of Obstetrics and Gynecology, West China Second University Hospital of Sichuan University, Chengdu, 610041 China; 2 Key Laboratory of Birth Defects and Related Diseases of Women and Children, Ministry of Education, Sichuan University, Chengdu, 610041 China

**Keywords:** Fetus, CNV: Copy number variation, Invasive prenatal diagnosis, CNL: Copy number loss, AD: Autosomal dominant, Gene, Prognosis

## Abstract

**Background:**

The broad application of high-resolution chromosome detection technology in prenatal diagnosis has identified copy number loss (CNL) involving autosomal dominant (AD) genes in certain fetuses. Exon sequencing of fetuses exhibiting structural anomalies yields diagnostic information in up to 20% of cases. However, there is currently no relevant literature about the genetic origin and pregnancy outcome of CNL involving AD genes in fetuses without structural abnormalities.

**Results:**

This was a prospective study involving pregnant women who underwent amniocentesis for fetal copy number variation sequencing (CNVseq). Detection of parent-of-origin was suggested in cases of samples with CNL involving AD genes and the pregnancy outcome was monitored. Amniotic fluid samples from 24,844 fetuses without structural abnormalities were successfully tested via CNVseq. The results showed that 134 fetuses (0.5%) had small CNL (< 10 Mb) containing AD genes, after excluding microdeletion and microduplication syndrome and polymorphisms. By monitoring the pregnancy outcomes of the 134 fetuses, we found that 104 (77.6%) were good, 13 (9.7%) were adverse, and 17 (12.7%) pregnant women voluntarily chose to terminate pregnancy. Of the 13 fetuses with adverse pregnancy outcomes, only 2 fetuses had phenotypes consistent with those of diseases caused by AD genes involved in CNL.

**Conclusions:**

The overall prognosis for fetuses without family history or structural abnormalities but with small CNL containing AD genes detected during pregnancy is good. The genetic origin, overlap status of established haploinsufficient gene and/or region, size of the CNL, and genetic mode may affect the pathogenicity of the CNL.

**Supplementary Information:**

The online version contains supplementary material available at 10.1186/s12864-022-08340-y.

## Background

Prenatal ultrasound is an established screening tool in obstetrics that provides increasingly high resolution to identify fetal structural abnormalities in approximately 5% of pregnancies [1]. The identification of chromosomal or genetic abnormalities is an important factor affecting fetal prognosis. Karyotyping and chromosomal microarray analysis (CMA) reveal that approximately 40% of these fetuses have genomic copy number variations (CNVs). However, close to 60% fail to receive a genomic diagnosis with which to inform prognosis and initiate genetic counseling [2]. In recent years, the implementation of prenatal exon sequencing (ES) in clinical practice to evaluate fetuses with structural anomalies has vastly improved the delineation of prognosis, providing clinical utility for a deeper understanding of the pathogenesis of prenatal genetic disorders. Prenatal ES in fetal structural anomalies yields diagnostic information in up to 20% of cases [3].

In some prenatal diagnostic centers, up to 74.8 – 96.8% of the fetuses undergoing invasive prenatal diagnosis did not exhibit structural abnormalities before amniocentesis [4–6]. Other indications for prenatal diagnosis include advanced maternal age, increased risk of a screening test, ultrasound soft markers, and maternal request; the main purpose is to detect genomic CNV, including aneuploidy and unbalanced chromosomal rearrangements. At present, high-resolution chromosome detection techniques, such as CMA and copy number variation sequencing (CNVseq), are widely used in fetal chromosome detection [7–12]. Compared with conventional karyotyping, CMA and CNVseq have an additional detection rate of 1–6% for clinically significant CNVs [6, 7, 13, 14] and also identify copy number loss (CNL) involving autosomal dominant (AD) genes.

For fetuses without ultrasound structural abnormalities, if high-resolution chromosome detection techniques indicate that there are CNL including AD genes in fetuses during pregnancy, what are their genetic origins and pregnancy outcomes? At present, such literature is lacking. This study sought to analyze the above contents to provide useful information for prenatal genetic counseling of mothers carrying fetuses with CNL.

## Results

**Fig. 1:**
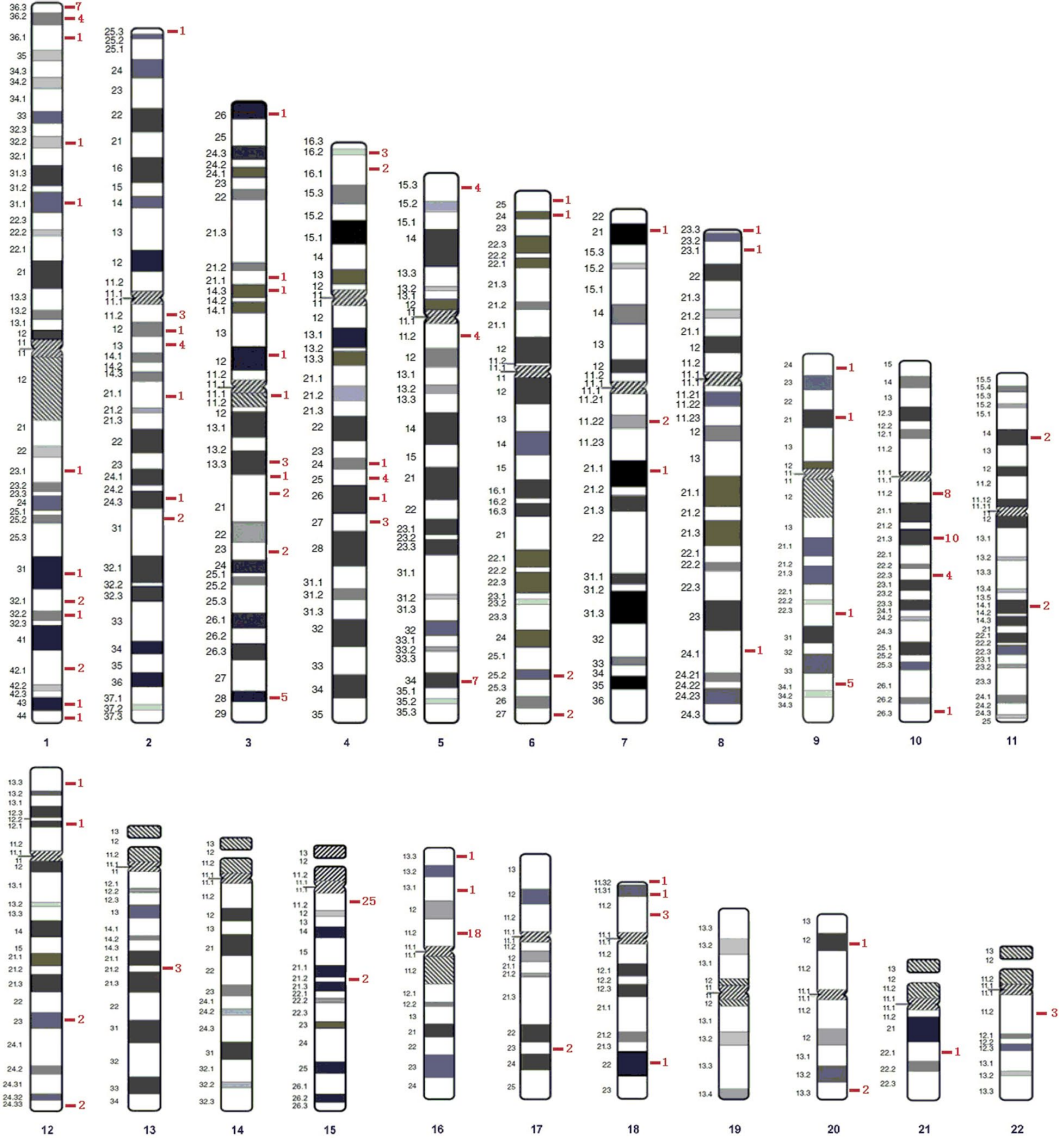
The genomic location of genes. The red Arabic numerals represent the number of genes located in the genome.

From February 2017 to June 2020, amniotic fluid samples from 24 844 fetuses without structural abnormalities were successfully analyzed via CNVseq. The results showed that 134 fetuses (0.5%) had CNL involving AD genes (< 10 Mb) after excluding microdeletion and microduplication syndrome (MMS) and polymorphisms; the average size of the CNL was 1.11 Mb, CNL involved a total of 202 AD genes, covering 128 different AD genes. Among the 134 samples, 37 carried two or more AD genes and one contained seven different AD genes. Among the 128 different genes, 23 (18.0%) were detected in multiple samples; *NIPA1* was the most common gene (25 samples), followed by *CTNNA3* (10 samples). According to the genomic location of genes (Fig. 1), these 128 genes were distributed across nearly all autosomes, with the highest number found on chromosome 15 (27 genes), followed by chromosomes 1 and 10 (23 genes, respectively); no CNL involving AD genes was found on chromosomes 14 and 19. The genomic positions, occurrences, genetic origins, and genetic modes of all 128 genes are summarized in the Supplementary Table.

Our follow-up on the pregnancy outcomes of the 134 fetuses found that 104 (77.6%) had good outcomes, 13 (9.7%) had adverse outcomes, and 17 (12.7%) pregnant women voluntarily chose termination of pregnancy (TOP). Details of the fetuses with adverse pregnancy outcomes are summarized in Table 1. CNL of the 134 samples were divided into inherited, de novo, and unknown according to their genetic origins. Then, the CNL overlap of established haploinsufficient (HI) gene and/or region (abbreviated as overlap of HI), average size of CNL, and outcome of pregnancy in each group were analyzed. The results showed that the number of samples that overlap of HI was the greatest, and the average size of CNL was the largest in the de novo group. Compared with the de novo group, the proportion of normal infants in the inherited group was higher (Pearson’s Chi-squared test, *P* = 0.000); the proportion of abnormal pregnancy outcomes was lower, but the difference was not statistically significant (Yates’ continuity correction of the Chi-squared test, *P* > 0.05). In the three groups, the rate of voluntary TOP in the de novo group was much higher than that in the other two groups (Yates’ continuity correction of the Chi-squared test, *P* = 0.000). These details are presented in Table 2.

**Table 1:** CNL involving AD genes: Information on 13 fetuses with adverse pregnancy outcomes

Case number	Indications of amniocentesis	Location	Size (Mb)	Gene	Phenotype and Inheritance	Origin	Pregnancy outcomes
A1	ultrasound soft markers	6p25.3p25.2	1.72	*FOXC1*	Anterior segment dysgenesis 3, multiple subtypes AD; Axenfeld-Rieger syndrome, type 3 AD	De novo	Facial abnormalities, Posterior fossa extraaxial cyst
A2	advanced maternal age	6q27	1.22	*ERMARD*	Periventricular nodular heterotopia 6 AD	Maternal	Ventricular septal defect 7 mm
A3	increased risk of a screening test	11q14.1q14.2	8.26	*ALG8;* *TENM4*	Congenital disorder of glycosylation, type Ih AR; Polycystic liver disease 3 with or without kidney cysts AD; Essential tremor, hereditary, 5 AD	Unknown	Multiple malformations
A4	ultrasound soft markers	10q21.3	0.38	*CTNNA3*	Arrhythmogenic right ventricular dysplasia, familial, 13 AD	Unknown	Duodenal stenosis and atresia
A5	increased risk of a screening test	4q25	0.22	*SGMS2*	Calvarial doughnut lesions with bone fragility with or without spondylometaphyseal dysplasia AD	Paternal	Hypospadias
A6	advanced maternal age	4q24	0.62	*PPP3CA*	Arthrogryposis, cleft palate, craniosynostosis, and impaired intellectual development AD; Developmental and epileptic encephalopathy 91 AD	Unknown	Hydronephrosis
A7	increased risk of a screening test	18q22.3q23	4.18	*TSHZ1*	Aural atresia, congenital AD	De novo	Developmental retardation
A8	advanced maternal age	11p15.1p14.3	3.26	*ANO5*	Gnathodiaphyseal dysplasia AD; Miyoshi muscular dystrophy 3 AR; Muscular dystrophy, limb-girdle, autosomal recessive 12 AR	De novo	Brain glioma
A9	increased risk of a screening test	11p14.3	0.16	*ANO5*	Gnathodiaphyseal dysplasia AD; Miyoshi muscular dystrophy 3 AR; Muscular dystrophy, limb-girdle, autosomal recessive 12 AR	Unknown	Multiple malformations
A10	advanced maternal age	5q11.2	0.22	*PDE4D*	Acrodysostosis 2, with or without hormone resistance AD	Unknown	Deaf
A11	ultrasound soft markers	3p26.1	0.34	*ITPR1*	Gillespie syndrome AD, AR; Spinocerebellar ataxia 15 AD; Spinocerebellar ataxia 29, congenital nonprogressive AD	Paternal	Short limbs
A12	increased risk of a screening test	15q11.2	0.32	*NIPA1*	Spastic paraplegia 6, autosomal dominant AD	Paternal	Spontaneous abortion
A13	increased risk of a screening test	20q13.33	0.14	*CHRNA4;* *KCNQ2*	{Nicotine addiction, susceptibility to}; Epilepsy, nocturnal frontal lobe, 1 AD; Developmental and epileptic encephalopathy 7 AD; Myokymia AD; Seizures, benign neonatal, 1 AD	Unknown	Epilepsy

*AD,* Autosomal dominant, *AR,*Autosomal recessive

**Table 2:** CNV information and pregnancy outcome of CNVs grouped by parent-of-origin

Group	Number of samples, *n* (%)	Overlap of HI, *n* (%)	Average size of CNV(Mb)	Outcome of pregnancy, *n* (%)		
				**Normal infants**	**Abnormal pregnancy outcomes**	**Voluntary TOP**
Detection of parent-of-origin	82 (61.2%)	25 (30.5%)	1.07	62 (75.6%)	7 (8.5%)	13 (15.9%)
Inherited	60 (73.2%)	15 (25.0%)	0.81	55 (91.7%)	4 (6.7%)	1 (1.7%)
De novo	22 (26.8%)	10 (45.5%)	1.77	7 (31.8%)	3 (13.6%)	12 (54.5%)
Unknown origin	52 (38.8%)	16 (30.8%)	1.18	42 (80.8%)	6 (11.5%)	4 (7.7%)
**Total**	134 (100.0%)	41 (30.6%)	1.11	104 (77.6%)	13 (9.7%)	17 (12.7%)

*CNV,* copynumbervariation; *HI*, Haploinsufficiency; *TOP*, termination of pregnancy

Based on content in the Online Mendelian Inheritance in Man® database (OMIM, https://omim.org/), we analyzed information regarding gene pathogenicity. Among the 128 different genes, 65 (50.8%) exhibited multiple genetic modes. A total of 202 AD genes were detected in 134 samples, which were divided into inherited, de novo, and unknown origin groups. Our results showed that, in the de novo group, the proportion of genes with multiple genetic modes was higher than that in the inherited group. The specific information is listed in Table 3.

**Table 3:** Information about gene pathogenicity

Group	One patient or family reported	Susceptibility	Somatic	ARorDD	Only AD
different genes (*n* = 128)	14 (10.9%)	18 (14.1%)	6 (4.7%)	40 (31.3%)	63 (49.2%)
Total number of genes (*n* = 202)	16 (7.9%)	22 (10.9%)	8 (4.0%)	51 (25.2%)	122 (60.4%)
Inherited (*n* = 81)	10 (12.3%)	9 (11.1%)	1 (1.2%)	19 (23.5%)	49 (60.5%)
De novo (*n* = 42)	2 (4.8%)	6 (14.3%)	4 (9.5%)	12 (28.6%)	22 (52.4%)
Unknown origin (*n* = 79)	4 (5.1%)	7 (8.9%)	3 (3.8%)	20 (25.3%)	51 (64.6%)

*AR,* Autosomal recessive, *DD*, Digenic dominant, *AD*, Autosomal dominant

CNL were divided into < 1 Mb, 1–3 Mb, and > 3 Mb groups according to fragment size. The results showed that the number of samples with CNL < 1 Mb accounted for the largest proportion (73.1%). The smaller the fragment, higher the proportion of normal pregnancy outcomes and lower the proportion of abnormal pregnancy outcomes, but there was no significant difference (Fisher’s exact test, Yates’ continuity correction of the Chi-squared test and Pearson’s Chi-squared test, P > 0.05). The relevant information is shown in Table 4.

**Table 4:** Information and pregnancy outcome of different sizes of CNVs

Size of CNV	Number of samples, n (%)	Average size of CNV (Mb)	Overlap of HI, n (%)	Outcome of pregnancy, *n* (%)		
				**Normal infants**	**Abnormal pregnancy outcomes**	**Voluntary TOP**
< 1 Mb	98 (73.1%)	0.39	37 (37.8%)	82 (83.7%)	8 (8.2%)	8 (8.2%)
1-3 Mb	20 (14.9%)	2.01	2 (10.0%)	13 (65.0%)	2 (10.0%)	5 (25.0%)
> 3 Mb	16 (11.9%)	4.46	2 (12.5%)	9 (56.3%)	3 (18.8%)	4 (25.0%)
Total	134 (100.0%)	1.11	41 (30.6%)	104 (77.6%)	13 (9.7%)	17 (12.7%)

*CNV,* copynumbervariation; *HI*, Haploinsufficiency; *TOP*, termination of pregnancy

## Discussion

In the nearly 60 years since prenatal diagnosis of genetic diseases was first proposed, the field has made great progress. While invasive fetal sampling technology has improved, the technological progress of cytogenetics and molecular biology has expanded the scope of genetic disease diagnosis even further. Presently, chorionic villus sampling and amniocentesis can be used to diagnose the majority of diseases with known genetic causes; moreover, the genomes and exomes of abnormal fetuses can be sequenced to help identify potential genetic vulnerabilities [15–19].

Theoretically, full gene, single-exon, or multi-exon deletion is often assumed to disrupt gene function by causing the complete absence of the gene product due to lack of transcription or nonsense-mediated decay of an altered transcript as identified by very strong evidence of pathogenicity in the ACMG/AMP guidelines [20]. However, our follow-up on the pregnancy outcomes of the 134 fetuses in this study found that 13 (9.7%) had abnormal phenotypes and only 2 had abnormal phenotypes consistent with the phenotypes of diseases caused by AD genes. Most of the women (77.6%) had good pregnancy outcomes, possibly because CNL in these subjects did not affect the functional region of the AD gene, partial exon deletion did not affect the protein integrity, loss of function was not the pathogenic mechanism of the disease, loss of function was at the 3’ terminal, or the gene had multiple transcripts [20–23]. In addition, certain autosomal dominant genetic diseases have incomplete penetrance, late onset age, and variable expressivity [24–26].

The results of this study showed that in the de novo group, the proportion of samples that overlap of HI was the highest, average size of the CNL was the biggest, and proportion of fetal adverse pregnancy outcome was the highest. At the same time, larger the CNL fragment, the higher the proportion of adverse pregnancy outcomes. Whether CNL overlaps the HI gene or region, the number of protein coding genes involved in genomic variation and genetic origin are important components of the pathogenicity classification of CNV [20, 27]. Generally, the larger the CNL fragment, the more protein coding genes involved. Therefore, if the CNL overlaps HI and the CNL fragment is large and de novo, the pathogenicity classification of CNL tends to be pathogenic or likely pathogenic. According to information provided in the OMIM database, in addition to AD inheritance, some genes may have other ways of causing diseases, such as disease susceptibility, somatic variation, autosomal recessive inheritance, or digenic inheritance. Some genes have been reported in only one patient or family, requiring further confirmation of the correlation with disease [28, 29]. The results of this study showed that, in the de novo group, the proportion of genes with multiple genetic modes was higher than that in the inherited group. In brief, the genetic origin of variation, overlap of HI, size of CNL, and genetic mode may affect the pathogenicity of CNL.

At present, neither CNVseq nor CMA technology can determine the accurate breakpoint location of CNVs [30]. Among 134 fetuses with CNL, 36 samples had CNL only partially covers an AD gene, we cannot identify the specific region of the AD gene covered by the CNL and whether it involves a coding/functional region; consequently, we cannot know whether this variation causes gene dysfunction and disease. However, if each sample is tested via other methods to determine the specific deletion region of the AD gene and the effect of gene function, this will undoubtedly increase the cost of detection and prolong the time required to obtain the report, leading to even greater anxiety in pregnant women. In this study, the biological parents of 82 fetuses were compared: among them, 60 fetuses (73.2%) inherited CNL from a parent with normal phenotype. Through follow-up, we determined that only 4 fetuses (6.7%) had abnormal pregnancy outcomes: A2, A5, A11, and A12. However, their phenotypes were not consistent with those of patients diagnosed with diseases caused by the AD genes involved in CNL. The CNL of 22 fetuses (26.8%) were de novo and 3 fetuses (13.6%) had adverse outcomes (A1, A7, and A8), with only the phenotype of A1 consistent with the AD gene involved in CNL. The genetic origin of CNL in 52 samples was unclear; 6 cases (11.5%) had adverse outcomes, with only the phenotype of one fetus (A13) associated with the disease caused by the AD gene involved in CNL. From the data shown in Table 2, we can see that compared with the unknown origin group, if the fetal CNL is inherited from the father or mother with normal phenotype, the proportion of normal pregnancy outcomes is higher and the proportion of voluntary TOP is lower. However, if the fetal CNL is de novo, the proportion of voluntary TOP is very high (12/22, 54.5%); we cannot know whether these fetuses would have had abnormal phenotypes, which may lead to the termination of many healthy fetuses. Therefore, in order to provide more reasonable medical advice—while saving time and lowering cost—it is recommended that the genetic origin of the CNL be confirmed to help evaluate its pathogenicity. If CNL is genetic in origin, an analysis of the pathogenicity of the AD gene by combining the phenotypes of parents and family members is warranted. In this study, the proportion of normal pregnancy outcomes in the inherited group was 91.7% (55/60). If the CNL is de novo, it is suggested that other methods be used to confirm the effect of the CNL on the function of the AD gene to help determine its pathogenicity, so as to provide more data-driven medical advice to pregnant women and their families.

## Conclusions

The overall prognosis for fetuses without family history or structural abnormalities but with small CNL containing AD genes (< 10 Mb) detected during pregnancy, after excluding MMS and polymorphism, is good. Protocols that combine the CNL data of parents with the results of AD gene function tests are critical to inform medical consultation and decision rules.

## Methods

### Study patients

This study involved pregnant women who underwent amniocentesis for fetal CNVseq during their second or third trimester due to reasons, such as advanced maternal age, increased risk of a screening test, ultrasound soft markers, or maternal request at West China Second University Hospital of Sichuan University from February 2017 to June 2020. Fetuses with structural abnormalities, based on the ultrasonogram before amniocentesis and family history of a genetic condition, were excluded.

### Amniotic fluid sample collection and DNA extraction

Based on routine collection procedures, 20 mL amniotic fluid was extracted and separated into 4 sterile centrifuge tubes containing 4 mL, 4 mL, 6 mL, and 6 mL, respectively. CNVseq was performed using the 6-mL amniotic fluid samples and quantitative fluorescence PCR (QF-PCR) was performed using one of the 4-mL amniotic fluid samples. The other two tubes of amniotic fluid were stored in a 2–8 ℃ refrigerator. According to the manufacturer's instructions, DNA was extracted from amniotic fluid samples using a DNeasy Blood and Tissue Kit (Qiagen, Hilden, Germany). QF-PCR was performed using 21 trisomy/sex chromosome/polyploidy and 18 trisomy/13 trisomy/polyploidy detection kits (DaAn Gene, Guangzhou, China). When QF-PCR results indicated the presence of maternal cells in the samples, CNVseq and QF-PCR were repeated on the spare samples after cell culture.

### CNVseq

DNA libraries were prepared using a Chromosome CNV Detection kit (Berry Genomics, Beijing, China) and subsequently sequenced on the Illumina NextSeq500 sequencing platform using a NextSeq500 High Output kit (Illumina, San Diego, CA, USA) according to the manufacturer's instructions. We compared the reads obtained by next generation sequencing with the GRCh37 reference genome and performed bioinformatics analysis to obtain the genomic copy number information of the samples as described previously [14]. In this study, the pathogenicity of CNVs > 100 kb was analyzed. The clinical significance of the CNVs was interpreted according to the technical standards for the interpretation and reporting of constitutional CNVs established by joint consensus of the American College of Medical Genetics and Genomics (ACMG) and the Clinical Genome Resource (ClinGen) [31]. After excluding MMS and polymorphisms (> 1% in the general population), CNL (the copy number is 1) involving AD genes with fragment sizes < 10 Mb were included in the present study. CNVs were confirmed using array-based comparative genomic hybridization (aCGH) or a repeat of CNVseq. aCGH was performed using a CGX v2 Oligo aCGH Kit (Agilent Technologies, Santa Clara, CA, USA). The microarray was scanned using the Agilent SureScan Microarray Scanner (Agilent). Data were extracted using the Agilent CytoGenomics software (Agilent) and analyzed using the Genoglyphix Analysis software (PerkinElmer, Waltham, MA, USA).

### Detection of parent-of-origin

When CNL involving AD genes was identified in the amniotic fluid sample, we recommended that the biological parents of the fetus undergo CNVseq to determine the origin of the fetal CNL; 2 mL of peripheral blood was collected from each parent and anticoagulated with EDTA. DNA extraction and CNVseq methods were performed as described for the amniotic fluid samples.

### Follow-up of pregnancy outcome

**Fig. 2:**
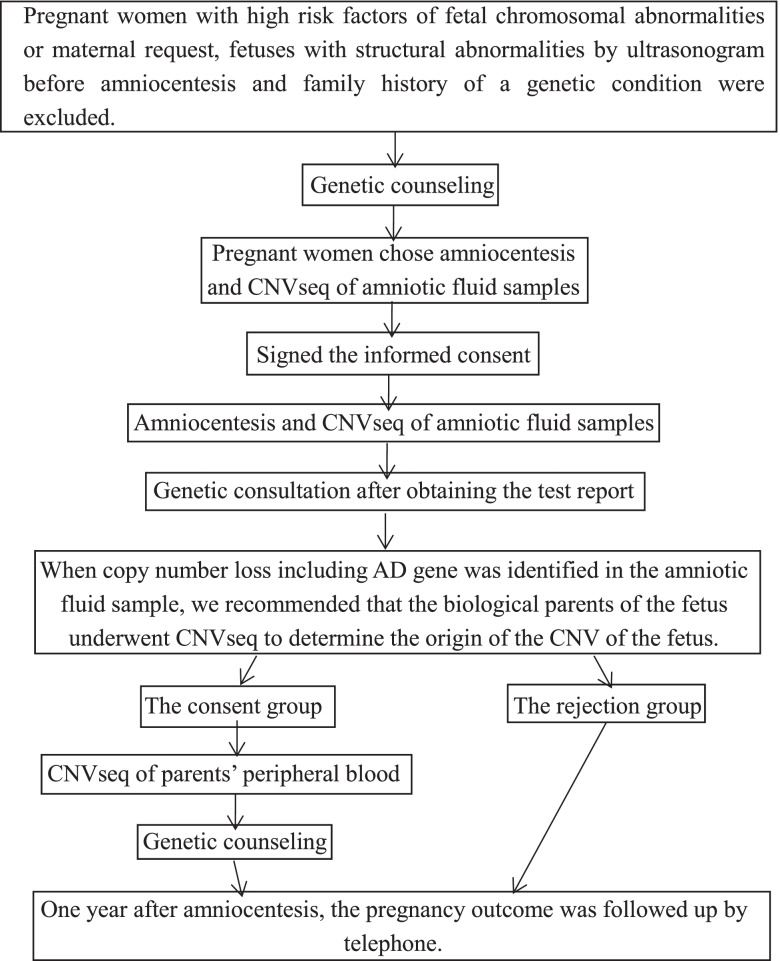
The flowchart of the study

One year after amniocentesis, the researchers contacted the mother or father of the fetus for follow-up. The information discussed during the inquiry included fetal ultrasound results; pregnancy complications; pregnancy loss; TOP and the causes; date and mode of delivery; weight and length of the newborn; the Apgar score; physical appearance of the newborn (i.e., normal/abnormal); feeding conditions after birth; and examination results of pediatric outpatient services. The flowchart of the study is shown in Fig. 2.

## Supplementary Information

**Additional file 1. **Supplementary Table: The genomic positions, occurrences, genetic origins, and genetic modes of all 128genes.

## Data Availability

The raw datasets used and analysed during the current study are not deposited in publicly available repositories because of considerations about the security of human genetic resources. The genetic mode and disease information of relevant genes in this article can be obtained from the OMIM database: https://www.omim.org/. For other details of the availability of data and material, please refer to the methods section of the article and Supplementary Table. Sequencing dataset can be obtained from the corresponding author on reasonable request.

## Abbreviations

CNL: Copy number loss
AD: Autosomal dominant
CNVseq: Copy number variation sequencing
CMA: Chromosomal microarray analysis
CNVs: Copy number variations
QF-PCR: Quantitative fluorescence PCR
ACMG: American College of Medical Genetics and Genomics
ClinGen: Clinical Genome Resource
MMS: Microdeletion and microduplication syndrome
aCGH: Array-based comparative genomic hybridization
TOP: Termination of pregnancy
