# Supplementary material for: Analysis of autosomal dominant genes impacted by copy number loss in 24,844 fetuses without structural abnormalities
Source: BMC Genomics. 2022 Feb 2;23:94. doi: 10.1186/s12864-022-08340-y (PMC8812209; doi:10.1186/s12864-022-08340-y)
Supplement: Supplementary file 1 — Additional file 1. Supplementary Table: The genomic positions, occurrences, genetic origins, and genetic modes of all 128genes. [file 12864_2022_8340_MOESM1_ESM.doc]

**Supplementary Table:** The genomic positions, occurrences, genetic origins, and genetic modes of all 128 genes

| **S/N** | **Gene** | **Location** | **Phenotype, Phenotype MIM number,Inheritance** | **n** | **Origin** | **Contained in CNL or Overlap** | **Others** |
| --- | --- | --- | --- | --- | --- | --- | --- |
| 1 | TNNI3K | 1p31.1 | Cardiac conduction disease with or without dilated cardiomyopathy 616117 AD | 1 | Paternal | Contained |  |
| 2 | DAB1 | 1p32.2 | Spinocerebellar ataxia 37 615945 AD | 1 | Unknown origin | Overlap |  |
| 3 | SDHB | 1p36.13 | Gastrointestinal stromal tumor 606764 AD, IC Mitochondrial complex II deficiency, nuclear type 4 619224 AR Paraganglioma and gastric stromal sarcoma 606864 Paragangliomas 4 115310 AD Pheochromocytoma 171300 AD | 1 | De nove | Contained | #※ |
| 4 | PIK3CD | 1p36.22 | Immunodeficiency 14 615513 AD | 2 | Maternal | Contained |  |
| 5 | KIF1B | 1p36.22 | {Neuroblastoma, susceptibility to, 1} 256700 AD, SMu Charcot-Marie-Tooth disease, type 2A1 118210 AD Pheochromocytoma 171300 AD | 1 | Maternal | Contained | * |
| 6 | RERE | 1p36.23 | Neurodevelopmental disorder with or without anomalies of the brain, eye, or heart 616975 AD | 1 | Maternal | Contained |  |
| 7 | PRDM16 | 1p36.32 | Cardiomyopathy, dilated, 1LL 615373 AD Left ventricular noncompaction 8 615373 AD | 1 | Unknown origin | Contained |  |
| 8 | ATAD3A | 1p36.33 | Harel-Yoon syndrome 617183 AD, AR Pontocerebellar hypoplasia, hypotonia, and respiratory insufficiency syndrome, neonatal lethal 618810 AR | 1 | Unknown origin | Contained | ※ |
| 9 | DVL1 | 1p36.33 | Robinow syndrome, autosomal dominant 2 616331 AD | 1 | Unknown origin | Contained |  |
| 10 | GABRD | 1p36.33 | {Epilepsy, generalized, with febrile seizures plus, type 5, susceptibility to} 613060 AD {Epilepsy, idiopathic generalized, 10} 613060 AD {Epilepsy, juvenile myoclonic, susceptibility to} 613060 AD | 1 | Unknown origin | Contained | * |
| 11 | GNB1 | 1p36.33 | Leukemia, acute lymphoblastic, somatic 613065 Mental retardation, autosomal dominant 42 616973 AD Myelodysplastic syndrome, somatic 614286 | 1 | Unknown origin | Contained | & |
| 12 | TMEM240 | 1p36.33 | Spinocerebellar ataxia 21 607454 AD | 1 | Unknown origin | Contained |  |
| 13 | SKI | 1p36.33-p36.32 | Shprintzen-Goldberg syndrome 182212 AD | 1 | Unknown origin | Contained |  |
| 14 | SPTA1 | 1q23.1 | Elliptocytosis-2 130600 AD Pyropoikilocytosis 266140 AR Spherocytosis, type 3 270970 AR | 1 | Maternal | Overlap | ※ |
| 15 | CDC73 | 1q31.2 | Hyperparathyroidism-jaw tumor syndrome 145001 AD Hyperparathyroidism, familial primary 145000 AD Parathyroid adenoma with cystic changes 145001 AD Parathyroid carcinoma 608266 | 1 | Maternal | Contained |  |
| 16 | CACNA1S | 1q32.1 | {Malignant hyperthermia susceptibility 5} 601887 AD {Malignant hyperthermia, susceptibility to, 5} 601887 AD {Thyrotoxic periodic paralysis, susceptibility to, 1} 188580 AD Hypokalemic periodic paralysis, type 1 170400 AD | 1 | De nove | Contained | * |
| 17 | INAVA | 1q32.1 | {Inflammatory bowel disease 29} 618077 AD | 1 | De nove | Contained | * |
| 18 | KCNH1 | 1q32.2 | Temple-Baraitser syndrome 611816 AD Zimmermann-Laband syndrome 1 135500 AD | 1 | De nove | Overlap |  |
| 19 | ACTA1 | 1q42.13 | ?Myopathy, scapulohumeroperoneal 616852 AD Myopathy, actin, congenital, with cores 161800 AD, AR Myopathy, actin, congenital, with excess of thin myofilaments 161800 AD, AR Myopathy, congenital, with fiber-type disproportion 1 255310 AD, AR Nemaline myopathy 3, autosomal dominant or recessive 161800 AD, AR | 1 | Maternal | Contained | #※ |
| 20 | GJC2 | 1q42.13 | Leukodystrophy, hypomyelinating, 2 608804 AR Lymphatic malformation 3 613480 AD Spastic paraplegia 44, autosomal recessive 613206 AR | 1 | Maternal | Contained | ※ |
| 21 | GREM2 | 1q43 | Tooth agenesis, selective, 9 617275 AD | 1 | Unknown origin | Contained |  |
| 22 | NLRP3 | 1q44 | CINCA syndrome 607115 AD Deafness, autosomal dominant 34, with or without inflammation 617772 AD Familial cold inflammatory syndrome 1 120100 AD Keratoendothelitis fugax hereditaria 148200 AD Muckle-Wells syndrome 191900 AD | 1 | Maternal | Contained |  |
| 23 | MYT1L | 2p25.3 | Mental retardation, autosomal dominant 39 616521 AD | 1 | Unknown origin | Contained |  |
| 24 | SNRNP200 | 2q11.2 | Retinitis pigmentosa 33 610359 AD | 1 | Unknown origin | Overlap |  |
| 25 | STARD7 | 2q11.2 | Epilepsy, familial adult myoclonic, 2 607876 AD | 1 | Unknown origin | Contained |  |
| 26 | TMEM127 | 2q11.2 | {Pheochromocytoma, susceptibility to} 171300 AD | 1 | Unknown origin | Contained | * |
| 27 | SLC5A7 | 2q12.3 | Myasthenic syndrome, congenital, 20, presynaptic 617143 AR Neuronopathy, distal hereditary motor, type VIIA 158580 AD | 1 | Maternal | Contained | ※ |
| 28 | EDAR | 2q13 | [Hair morphology 1, hair thickness] 612630 Ectodermal dysplasia 10A, hypohidrotic/hair/nail type, autosomal dominant 129490 AD Ectodermal dysplasia 10B, hypohidrotic/hair/tooth type, autosomal recessive 224900 AR | 2 | Maternal(1), Unknown origin(1) | Contained | ※ |
| 29 | RANBP2 | 2q13 | {Encephalopathy, acute, infection-induced, 3, susceptibility to} 608033 AD | 2 | Maternal(1), Unknown origin(1) | Contained | * |
| 30 | TUBA3D | 2q21.1 | Keratoconus 9 617928 AD | 1 | Unknown origin | Contained |  |
| 31 | SCN9A | 2q24.3 | Erythermalgia, primary 133020 AD Insensitivity to pain, congenital 243000 AR Neuropathy, hereditary sensory and autonomic, type IID 243000 AR Paroxysmal extreme pain disorder 167400 AD Small fiber neuropathy 133020 AD | 1 | Maternal | Overlap | ※ |
| 32 | CHN1 | 2q31.1 | Duane retraction syndrome 2 604356 AD | 1 | De nove | Contained |  |
| 33 | CHRNA1 | 2q31.1 | Multiple pterygium syndrome, lethal type 253290 AR Myasthenic syndrome, congenital, 1A, slow-channel 601462 AD Myasthenic syndrome, congenital, 1B, fast-channel 608930 AD, AR | 1 | De nove | Contained | ※ |
| 34 | ROBO2 | 3p12.3 | Vesicoureteral reflux 2 610878 AD | 1 | Paternal | Overlap |  |
| 35 | WNT5A | 3p14.3 | Robinow syndrome, autosomal dominant 1 180700 AD | 1 | Unknown origin | Contained |  |
| 36 | CACNA1D | 3p21.1 | Primary aldosteronism, seizures, and neurologic abnormalities 615474 AD Sinoatrial node dysfunction and deafness 614896 AR | 1 | Unknown origin | Contained | ※ |
| 37 | ITPR1 | 3p26.1 | Gillespie syndrome 206700 AD, AR Spinocerebellar ataxia 15 606658 AD Spinocerebellar ataxia 29, congenital nonprogressive 117360 AD | 1 | Paternal | Overlap | ※ |
| 38 | PROS1 | 3q11.1 | Thrombophilia due to protein S deficiency, autosomal dominant 612336 AD Thrombophilia due to protein S deficiency, autosomal recessive 614514 AR | 1 | Maternal | Contained | ※ |
| 39 | ARHGAP31 | 3q13.32-q13.33 | Adams-Oliver syndrome 1 100300 AD | 1 | Maternal | Contained |  |
| 40 | C3ORF9 | 3q13.33 | ?Muscular dystrophy, limb-girdle, autosomal recessive 21 617232 AR Dowling-Degos disease 4 615696 AD | 1 | Maternal | Contained | #※ |
| 41 | RABL3 | 3q13.33 | {?Pancreatic cancer, susceptibility to, 5} 618680 AD | 1 | Maternal | Contained | #* |
| 42 | CASR | 3q13.33-q21.1 | Hyperparathyroidism, neonatal 239200 AD, AR Hypocalcemia, autosomal dominant 601198 AD Hypocalcemia, autosomal dominant, with Bartter syndrome 601198 AD Hypocalciuric hypercalcemia, type I 145980 AD | 1 | Maternal | Contained | ※ |
| 43 | ADCY5 | 3q21.1 | Dyskinesia, familial, with facial myokymia 606703 AD | 1 | Maternal | Contained |  |
| 44 | MLCK | 3q21.1 | Aortic aneurysm, familial thoracic 7 613780 AD | 1 | Maternal | Contained |  |
| 45 | ATR | 3q23 | ?Cutaneous telangiectasia and cancer syndrome, familial 614564 AD Seckel syndrome 1 210600 AR | 1 | Paternal | Contained | # |
| 46 | PLS1 | 3q23 | Deafness, autosomal dominant 76 618787 AD | 1 | Paternal | Overlap |  |
| 47 | LPP | 3q27.3-q28 | Leukemia, acute myeloid 601626 AD, SMu Lipoma | 1 | De nove | Contained | & |
| 48 | CCDC50 | 3q28 | ?Deafness, autosomal dominant 44 607453 AD | 2 | Maternal(1), De nove(1) | Contained | # |
| 49 | TP63 | 3q28 | ADULT syndrome 103285 AD Ectrodactyly, ectodermal dysplasia, and cleft lip/palate syndrome 3 604292 AD Hay-Wells syndrome 106260 AD Limb-mammary syndrome 603543 AD Orofacial cleft 8 618149 Rapp-Hodgkin syndrome 129400 AD Split-hand/foot malformation 4 605289 AD | 1 | De nove | Contained |  |
| 50 | FGF12 | 3q28-q29 | Developmental and epileptic encephalopathy 47 617166 AD | 1 | De nove | Contained |  |
| 51 | DRD5 | 4p16.1 | {Attention deficit-hyperactivity disorder, susceptibility to} 143465 AD {Blepharospasm, primary benign} 606798 AD | 1 | Paternal | Contained | * |
| 52 | GLUT9 | 4p16.1 | {Uric acid concentration, serum, QTL 2} 612076 AD, AR  Hypouricemia, renal, 2 612076 AD, AR | 1 | Paternal | Contained | *※ |
| 53 | EVC2 | 4p16.2 | Ellis-van Creveld syndrome 225500 AR Weyers acrofacial dysostosis 193530 AD | 2 | Unknown origin | Overlap | ※ |
| 54 | EVC | 4p16.2 | ?Weyers acrofacial dysostosis 193530 AD Ellis-van Creveld syndrome 225500 AR | 1 | Unknown origin | Contained | #※ |
| 55 | PPP3CA | 4q24 | Arthrogryposis, cleft palate, craniosynostosis, and impaired intellectual development 618265 AD Developmental and epileptic encephalopathy 91 617711 AD | 1 | Unknown origin | Overlap |  |
| 56 | SGMS2 | 4q25 | Calvarial doughnut lesions with bone fragility with or without spondylometaphyseal dysplasia 126550 AD | 4 | Paternal | Contained |  |
| 57 | MYOZ2 | 4q26 | Cardiomyopathy, hypertrophic, 16 613838 AD | 1 | Unknown origin | Overlap |  |
| 58 | ANXA5 | 4q27 | {Pregnancy loss, recurrent, susceptibility to, 3} 614391 AD | 1 | Unknown origin | Contained | * |
| 59 | NDNF | 4q27 | Hypogonadotropic hypogonadism 25 with anosmia 618841 AD | 1 | Unknown origin | Contained |  |
| 60 | TRPC3 | 4q27 | ?Spinocerebellar ataxia 41 616410 AD | 1 | Unknown origin | Contained | # |
| 61 | SDHA | 5p15.33 | Cardiomyopathy, dilated, 1GG 613642 AR Mitochondrial complex II deficiency, nuclear type 1 252011 AR Paragangliomas 5 614165 AD | 2 | De nove(1), Unknown origin(1) | Contained | ※ |
| 62 | SLC6A19 | 5p15.33 | Hartnup disorder 234500 AR Hyperglycinuria 138500 AD Iminoglycinuria, digenic 242600 AR, DR | 1 | De nove | Contained | ※ |
| 63 | TERT | 5p15.33 | {Dyskeratosis congenita, autosomal dominant 2} 613989 AD, AR {Dyskeratosis congenita, autosomal recessive 4} 613989 AD, AR {Leukemia, acute myeloid} 601626 AD, SMu {Melanoma, cutaneous malignant, 9} 615134 {Pulmonary fibrosis and/or bone marrow failure, telomere-related, 1} 614742 AD | 1 | De nove | Contained | *※ |
| 64 | PDE4D | 5q11.2 | Acrodysostosis 2, with or without hormone resistance 614613 AD | 3 | Maternal(1), Unknown origin(2) | Overlap |  |
| 65 | HSPB3 | 5q11.2 | ?Neuronopathy, distal hereditary motor, type IIC 613376 AD | 1 | Unknown origin | Contained | # |
| 66 | GABRA1 | 5q34 | {Epilepsy, childhood absence, susceptibility to, 4} 611136 {Epilepsy, juvenile myoclonic, susceptibility to, 5} 611136 Developmental and epileptic encephalopathy 19 615744 AD | 2 | Maternal(1), De nove(1) | Contained | * |
| 67 | GABRB2 | 5q34 | Developmental and epileptic encephalopathy 92 617829 AD | 2 | Maternal(1), De nove(1) | Contained |  |
| 68 | GABRG2 | 5q34 | Developmental and epileptic encephalopathy 74 618396 AD Epilepsy, generalized, with febrile seizures plus, type 3 607681 AD Febrile seizures, familial, 8 607681 AD | 2 | Maternal(1), De nove(1) | Contained |  |
| 69 | HMMR | 5q34 | {Breast cancer, susceptibility to} 114480 AD, SMu | 1 | Maternal | Contained | * |
| 70 | EDN1 | 6p24.1 | Auriculocondylar syndrome 3 615706 AR Question mark ears, isolated 612798 AD | 1 | Maternal | Contained | ※ |
| 71 | FOXC1 | 6p25.3 | Anterior segment dysgenesis 3, multiple subtypes 601631 AD Axenfeld-Rieger syndrome, type 3 602482 AD | 1 | De nove | Contained |  |
| 72 | ESR1 | 6q25.1-q25.2 | {Migraine, susceptibility to} 157300 AD {Myocardial infarction, susceptibility to} 608446 Breast cancer, somatic 114480 Estrogen resistance 615363 AR | 1 | Paternal | Overlap | *&※ |
| 73 | SYNE1 | 6q25.2 | Arthrogryposis multiplex congenita 3, myogenic type 618484 AR Emery-Dreifuss muscular dystrophy 4, autosomal dominant 612998 AD Spinocerebellar ataxia, autosomal recessive 8 610743 AR | 1 | Paternal | Overlap | ※ |
| 74 | ERMARD | 6q27 | ?Periventricular nodular heterotopia 6 615544 AD | 2 | Paternal(1), Maternal(1) | Contained | # |
| 75 | TMEM106B | 7p21.3 | Leukodystrophy, hypomyelinating, 16 617964 AD | 1 | Unknown origin | Contained |  |
| 76 | AUTS2 | 7q11.22 | Mental retardation, autosomal dominant 26 615834 AD | 2 | Maternal | Overlap |  |
| 77 | SEMA3E | 7q21.11 | ?CHARGE syndrome 214800 AD | 1 | Maternal | Overlap | # |
| 78 | CTSB | 8p23.1 | Keratolytic winter erythema 148370 AD | 1 | Unknown origin | Contained |  |
| 79 | ARHGEF10 | 8p23.3 | ?Slowed nerve conduction velocity, AD 608236 AD | 1 | Paternal | Contained | # |
| 80 | SAMD12 | 8q24.12 | Epilepsy, familial adult myoclonic, 1 601068 AD | 1 | Unknown origin | Overlap |  |
| 81 | C9orf72 | 9p21.2 | Frontotemporal dementia and/or amyotrophic lateral sclerosis 1 105550 AD | 1 | De nove | Contained |  |
| 82 | JAK2 | 9p24.1 | {Budd-Chiari syndrome, somatic} 600880 Erythrocytosis, somatic 133100 Leukemia, acute myeloid, somatic 601626 Myelofibrosis, somatic 254450 Polycythemia vera, somatic 263300 Thrombocythemia 3 614521 AD, SMu | 1 | De nove | Contained | *& |
| 83 | SPTLC1 | 9q22.31 | Neuropathy, hereditary sensory and autonomic, type IA 162400 AD | 1 | Unknown origin | Contained |  |
| 84 | ABL1 | 9q34.12 | Congenital heart defects and skeletal malformations syndrome 617602 AD Leukemia, Philadelphia chromosome-positive, resistant to imatinib 608232 SMu | 1 | De nove | Contained | & |
| 85 | CEL | 9q34.13 | Maturity-onset diabetes of the young, type VIII 609812 AD | 1 | De nove | Contained |  |
| 86 | GFI1B | 9q34.13 | Bleeding disorder, platelet-type, 17 187900 AD, AR | 1 | De nove | Contained | ※ |
| 87 | SETX | 9q34.13 | Amyotrophic lateral sclerosis 4, juvenile 602433 AD Spinocerebellar ataxia, autosomal recessive, with axonal neuropathy 2 606002 AR | 1 | De nove | Contained | ※ |
| 88 | TSC1 | 9q34.13 | Focal cortical dysplasia, type II, somatic 607341 Lymphangioleiomyomatosis 606690 Tuberous sclerosis-1 191100 AD | 1 | De nove | Contained | & |
| 89 | GDF2 | 10q11.22 | Telangiectasia, hereditary hemorrhagic, type 5 615506 AD | 5 | Maternal(1), Unknown origin(4) | Contained |  |
| 90 | ERCC6 | 10q11.23 | {Lung cancer, susceptibility to} 211980 AD, SMu {Macular degeneration, age-related, susceptibility to, 5} 613761 Cerebrooculofacioskeletal syndrome 1 214150 AR Cockayne syndrome, type B 133540 AR De Sanctis-Cacchione syndrome 278800 AR Premature ovarian failure 11 616946 AD UV-sensitive syndrome 1 600630 AR | 3 | Maternal(1), Unknown origin(2) | Contained | *※ |
| 91 | CTNNA3 | 10q21.3 | Arrhythmogenic right ventricular dysplasia, familial, 13 615616 AD | 10 | Paternal(1), Maternal(6), Unknown origin(3) | Overlap |  |
| 92 | ANXA11 | 10q22.3 | Amytrophic lateral sclerosis 23 617839 AD | 2 | Paternal(1), Maternal(1) | Overlap |  |
| 93 | NUTM2B-AS1 | 10q22.3 | ?Oculopharyngeal myopathy with leukoencephalopathy 1 618637 AD | 1 | Paternal | Contained | # |
| 94 | SFTPA2 | 10q22.3 | Pulmonary fibrosis, idiopathic 178500 AD | 1 | Paternal | Overlap |  |
| 95 | EBF3 | 10q26.3 | Hypotonia, ataxia, and delayed development syndrome 617330 AD | 1 | De nove | Contained |  |
| 96 | ANO5 | 11p14.3 | Gnathodiaphyseal dysplasia 166260 AD Miyoshi muscular dystrophy 3 613319 AR Muscular dystrophy, limb-girdle, autosomal recessive 12 611307 AR | 2 | De nove(1), Unknown origin(1) | Contained | ※ |
| 97 | ALG8 | 11q14.1 | Congenital disorder of glycosylation, type Ih 608104 AR Polycystic liver disease 3 with or without kidney cysts 617874 AD | 1 | Unknown origin | Contained | ※ |
| 98 | TENM4 | 11q14.1 | Essential tremor, hereditary, 5 616736 AD | 1 | Unknown origin | Contained |  |
| 99 | SOX5 | 12p12.1 | Lamb-Shaffer syndrome 616803 AD | 1 | Maternal | Overlap |  |
| 100 | VWF | 12p13.31 | von Willebrand disease, type 1 193400 AD von Willebrand disease, type 3 277480 AR von Willebrand disease, types 2A, 2B, 2M, and 2N 613554 AD, AR | 1 | Maternal | Overlap | ※ |
| 101 | MYBPC1 | 12q23.2 | Arthrogryposis, distal, type 1B 614335 AD  Lethal congenital contracture syndrome 4 614915 AR Myopathy, congenital, with tremor 618524 AD | 1 | Maternal | Contained | ※ |
| 102 | SYCP3 | 12q23.2 | Pregnancy loss, recurrent, 4 270960 AD Spermatogenic failure 4 270960 AD | 1 | Maternal | Contained |  |
| 103 | P2RX2 | 12q24.33 | Deafness, autosomal dominant 41 608224 AD | 1 | De nove | Contained |  |
| 104 | POLE | 12q24.33 | {Colorectal cancer, susceptibility to, 12} 615083 AD FILS syndrome 615139 AR IMAGE-I syndrome 618336 AR | 1 | De nove | Contained | *※ |
| 105 | DIAPH3 | 13q21.2 | Auditory neuropathy, autosomal dominant, 1 609129 AD | 3 | Paternal(2), Maternal(1) | Overlap |  |
| 106 | NIPA1 | 15q11.2 | Spastic paraplegia 6, autosomal dominant 600363 AD | 25 | Paternal(7), Maternal(5), De nove(3), Unknown origin(10) | Contained |  |
| 107 | AP4E1 | 15q21.2 | Spastic paraplegia 51, autosomal recessive 613744 AR Stuttering, familial persistent, 1 184450 AD | 1 | Paternal | Contained | ※ |
| 108 | CYP19A1 | 15q21.2 | Aromatase deficiency 613546 Aromatase excess syndrome 139300 AD | 1 | Paternal | Contained |  |
| 109 | KIF22 | 16p11.2 | Spondyloepimetaphyseal dysplasia with joint laxity, type 2 603546 AD | 6 | De nove(3), Unknown origin(3) | Contained |  |
| 110 | PRRT2 | 16p11.2 | Convulsions, familial infantile, with paroxysmal choreoathetosis 602066 AD Episodic kinesigenic dyskinesia 1 128200 AD Seizures, benign familial infantile, 2 605751 AD | 6 | De nove(3), Unknown origin(3) | Contained |  |
| 111 | TBX6 | 16p11.2 | Spondylocostal dysostosis 5 122600 AD, AR | 6 | De nove(3), Unknown origin(3) | Contained | ※ |
| 112 | PARN | 16p13.13 | Dyskeratosis congenita, autosomal recessive 6 616353 AR Pulmonary fibrosis and/or bone marrow failure, telomere-related, 4 616371 AD | 1 | Unknown origin | Contained | ※ |
| 113 | MEFV | 16p13.3 | Familial Mediterranean fever, AD 134610 AD Familial Mediterranean fever, AR 249100 AR Neutrophilic dermatosis, acute febrile 608068 AD | 1 | Maternal | Contained | ※ |
| 114 | GH1 | 17q23.3 | Growth hormone deficiency, isolated, type IA 262400 AR Growth hormone deficiency, isolated, type IB 612781 Growth hormone deficiency, isolated, type II 173100 AD Kowarski syndrome 262650 AR | 1 | Unknown origin | Contained | ※ |
| 115 | SCN4A | 17q23.3 | Hyperkalemic periodic paralysis, type 2 170500 AD Hypokalemic periodic paralysis, type 2 613345 AD Myasthenic syndrome, congenital, 16 614198 AR Myotonia congenita, atypical, acetazolamide-responsive 608390 AD Paramyotonia congenita 168300 AD | 1 | Unknown origin | Contained | ※ |
| 116 | GNAL | 18p11.21 | Dystonia 25 615073 AD | 1 | Unknown origin | Contained |  |
| 117 | APCDD1 | 18p11.22 | Hypotrichosis 1 605389 AD | 1 | Unknown origin | Contained |  |
| 118 | PIEZO2 | 18p11.22-p11.21 | ?Marden-Walker syndrome 248700 AD Arthrogryposis, distal, type 3 114300 AD Arthrogryposis, distal, type 5 108145 AD Arthrogryposis, distal, with impaired proprioception and touch 617146 AR | 1 | Unknown origin | Contained | #※ |
| 119 | TGIF1 | 18p11.31 | Holoprosencephaly 4 142946 AD | 1 | De nove | Contained |  |
| 120 | SMCHD1 | 18p11.32 | Bosma arhinia microphthalmia syndrome 603457 AD Fascioscapulohumeral muscular dystrophy 2, digenic 158901 DD | 1 | Unknown origin | Contained | ※ |
| 121 | TSHZ1 | 18q22.3 | Aural atresia, congenital 607842 AD | 1 | De nove | Contained |  |
| 122 | FLRT3 | 20p12.1 | Hypogonadotropic hypogonadism 21 with anosmia 615271 AD | 1 | Unknown origin | Contained |  |
| 123 | CHRNA4 | 20q13.33 | {Nicotine addiction, susceptibility to} 188890 Epilepsy, nocturnal frontal lobe, 1 600513 AD | 1 | Unknown origin | Contained | * |
| 124 | KCNQ2 | 20q13.33 | Developmental and epileptic encephalopathy 7 613720 AD Myokymia 121200 AD Seizures, benign neonatal, 1 121200 AD | 1 | Unknown origin | Contained |  |
| 125 | SON | 21q22.11 | ZTTK syndrome 617140 AD | 1 | Unknown origin | Overlap |  |
| 126 | LZTR1 | 22q22.11 | Schwannomatosis-2, susceptibility to} 615670 AD Noonan syndrome 10 616564 AD Noonan syndrome 2 605275 AR | 1 | Unknown origin | Contained | ※ |
| 127 | SERPIND1 | 22q22.11 | Thrombophilia due to heparin cofactor II deficiency 612356 AD | 1 | Unknown origin | Contained |  |
| 128 | CRYBB2 | 22q11.23 | Cataract 3, multiple types 601547 AD | 1 | Unknown origin | Overlap |  |
| n, occurrences of the gene; *#* One patient reported; *** Susceptibility; *&* Somatic; *※* autosomal recessive or digenic dominantl. | | | | | | | |
